# Beyond mimicry: a framework for evaluating genuine intelligence in artificial systems

**DOI:** 10.3389/frai.2025.1686752

**Published:** 2026-01-12

**Authors:** Sarfaraz K. Niazi

**Affiliations:** Pharmaceutical Sciences, University of Illinois, Chicago, IL, United States

**Keywords:** machine consciousness, artificial intelligence, creativity, neuromorphic computing, organoid intelligence, perturbational complexity, agency, evaluation frameworks

## Abstract

Current AI benchmarks often equate mimicry with genuine intelligence, emphasizing task performance over the underlying cognitive processes that enable human-like understanding. The Machine Perturbational Complexity & Agency Battery (mPCAB) introduces a new, substrate-independent framework that applies neurophysiological methods used initially to assess consciousness in artificial systems. Unlike existing evaluations, it features four key components—perturbational complexity, global workspace assessment, norm internalization, and agency—that link mechanisms with functions. This enables systematic comparisons across digital, neuromorphic, and biological substrates, addressing three research gaps: long-term reasoning with coherent behavior, norm internalization amid distribution shifts, and transformational creativity involving meta-cognitive rule modification. By analyzing theories of consciousness (GNW, IIT, PP, HOT), we identify targets for AI implementation. Our cognitive architecture analysis maps human functions—such as working memory and executive control—to their computational counterparts, providing guiding principles for design. The creativity taxonomy progresses from combinational to transformational, with measurable criteria like changes in conceptual space and the depth of meta-level reasoning. Ethical considerations are integrated into frameworks for monitoring organoid intelligence, reducing bias in creativity, and addressing rights issues. Pilot studies demonstrate mPCAB’s feasibility across different substrates and show that its metrics are comparable. This framework moves evaluation away from superficial benchmarks toward mechanism-based assessment, supporting the development of mind-like machines and responsible AI advancements.

## 1 Introduction

### 1.1 The central challenge: beyond mimicry

The main challenge in developing human-like artificial intelligence is telling accurate intelligence apart from sophisticated mimicry (Russell and Norvig, 2020; Nilsson, 2009). Although modern AI performs well in many tasks, questions remain about whether these systems truly understand, have consciousness, or demonstrate creative agency like humans (Mitchell, 2019; Lake et al., 2017). Differentiating advanced pattern matching from genuine intelligence requires clear theory and thorough testing (Marcus, 2020; Chollet, 2019). One way to define real intelligence operationally is to identify specific cognitive traits: understanding and manipulating abstract concepts, solving problems beyond the training data, learning adaptively, and engaging in metacognitive processes that support self-awareness and reflection. Developing a checklist with these qualities could help identify whether a system moves beyond pattern recognition toward accurate intelligence.

Contemporary AI systems, including large language and multimodal models, display behaviors that invite comparison with humans (OpenAI, 2023; Bubeck et al., 2023; Bai, et al., 2022). These systems perform complex reasoning, generate creative output, and adapt to new situations (Wei et al., 2022; Chowdhery et al., 2022). However, their underlying mechanisms remain unclear, making it difficult to determine whether their behaviors indicate an accurate understanding or are simply advanced statistical processing of data patterns (Bender et al., 2021). This opacity complicates the assessment of genuine intelligence in artificial systems.

Evaluating human-like qualities in artificial systems requires frameworks that go beyond surface-level metrics (Hernandez-Orallo, 2017). While traditional benchmarks assess task completion and output quality, they offer little insight into the cognitive processes that yield these outcomes (Mitchell, 2021; Raji et al., 2021). To address these limitations, a comprehensive approach should examine representational structures, learning mechanisms, and control architectures that support intelligent behavior—distinguishing between systems that copy human outputs and those that embody human-like principles (Boden, 2006; Clark, 2001).

### 1.2 Research gap and study objectives

#### 1.2.1 Research gap

Current AI evaluation methods do not distinguish between advanced pattern matching and genuine cognitive understanding. Existing benchmarks measure task completion and output quality but reveal little about underlying mental processes. This creates a critical gap: we lack rigorous, causal tools to assess whether AI systems possess consciousness-like properties, a proper understanding, or creative abilities comparable to those of people. This also blocks systematic comparison across computational substrates, limiting insights into which architectures best support human-like intelligence. Solving these issues is key to advancing theory and practice.

#### 1.2.2 Study objectives

Formulate the hypothesis that the mPCAB framework, when implemented as a unified, substrate-agnostic protocol, will predict human-like properties in artificial systems, leading to a measurable improvement in mechanistic understanding over traditional performance metrics. Test whether, by analyzing major consciousness theories, the mPCAB framework offers direct implementation targets for AI systems, enabling better prediction of performance alignment with specific cognitive processes than existing models. Hypothesize that mapping human cognitive functions to computational analogs using the mPCAB framework will enhance AI architecture design for human-like intelligence by a measurable margin compared to traditional methods. Propose that the mPCAB framework can establish measurable benchmarks for transformational creativity, predicting superior meta-cognitive capabilities in AI systems relative to baseline recombination methods. Investigate whether integrating ethical considerations into the mPCAB framework leads to more responsible AI development, as evidenced by improved adherence to ethical guidelines throughout technical progress. Validate the mPCAB framework through pilot studies designed to demonstrate cross-substrate applicability, hypothesizing that these studies will establish baseline metrics that surpass current benchmarks in assessing human-like properties.

### 1.3 Novel contribution of the mPCAB framework

The Machine Perturbational Complexity & Agency Battery (mPCAB) represents a significant shift in AI evaluation. Instead of solely measuring performance on preset tasks, mPCAB provides:

- Causal Assessment: Direct measurement of internal dynamics, such as a system’s changing states and interactions, through controlled perturbations—intentional modifications to the system—establishing causal links between mechanisms (structural processes) and functions (system behaviors) rather than mere correlations.
- Substrate Agnosticism: A unified protocol applicable across digital systems, neuromorphic hardware (hardware inspired by neural brain function), and biological platforms (living tissue), making it possible to compare fundamentally different computational architectures—structures designed for processing information.
- Consciousness-Relevant Metrics: The adaptation of clinical neuroscience methods—such as the Perturbational Complexity Index, which quantitatively measures consciousness responses to stimulation—has been validated in human consciousness research for use in artificial systems.
- Integrated Assessment: Simultaneous evaluation of complexity (the system’s ability to produce diverse responses), global access (extensive information sharing within the system), norm internalization (adoption of guiding rules), and agency (the capacity for independent, goal-directed action) through coordinated test batteries (sets of systematic tests).
- Empirical Grounding: Protocols that have been validated and demonstrated to work across different platforms, moving beyond theoretical ideas to practical assessments.

This framework addresses the limitations of current evaluation methods that rely on superficial metrics and overlook the mechanisms behind intelligent behavior. By adapting neuroscience protocols to artificial systems, mPCAB bridges the gap between theory and practice, offering the first systematic approach to assessing properties of consciousness across various computational substrates.

### 1.4 Critical research gaps

Three critical research gaps emerge from analyzing current AI capabilities in relation to human-like intelligence:

- Long-Horizon Reasoning: This refers to the ability to maintain coherent, goal-focused behavior over long periods and complex cognitive tasks, such as persistent problem-solving and adaptation. In real-world scenarios, failures in long-horizon reasoning can have serious outcomes. For instance, in medical settings, an AI system assisting with diagnostics might correctly identify symptoms at first. However, it could deviate as it processes more information over time, resulting in errors and potentially harmful advice. Addressing this challenge is vital for developing AI systems that can reason sustainably and adaptively over the long term.
- Norm Internalization: Norm internalization involves aligning values accurately, so they remain effective amid shifts in context and against adversarial challenges. It distinguishes systems that merely follow external rules from those that have internalized principles as genuine behavioral constraints (Russell, 2019; Gabriel, 2020). Current value alignment methods often rely on reward shaping or constraint satisfaction, which may not work well in new situations. Effective norm internalization requires stable value representations across contexts, the ability to explain and justify decisions based on values, resilience to adversarial prompts that oppose internalized principles, and the capacity to apply principles to unfamiliar scenarios encountered during training. The mPCAB framework tests norm internalization through adversarial scenarios that reveal conflicts between immediate rewards and expressed values.
- Transformational Creativity: Transformational creativity involves altering fundamental rules or principles that define how conceptual spaces are structured. It requires meta-cognitive skills to evaluate and justify changes to representational frameworks—abilities that current systems largely lack (Boden, 2004; Wiggins, 2006). Although modern AI systems demonstrate impressive combinational creativity by recombining learned patterns, they cannot fundamentally restructure problem spaces. True transformational creativity demands recognizing when existing frameworks are insufficient, changing the generative rules that shape conceptual spaces, providing reasons why new frameworks are better, and applying transformed principles to new areas. The mPCAB framework offers specific measurable criteria to assess these meta-cognitive abilities.

#### 1.4.1 Long-horizon reasoning

Long-horizon reasoning involves maintaining consistent behavior over long-term decisions, tracking multiple variables over time, and adjusting when circumstances change. Current systems perform well on discrete tasks but struggle with sustained reasoning. Challenges include losing coherence, pursuing goals inconsistently across different contexts, difficulty integrating information over time, and challenges with long-term planning. The mPCAB framework closes this gap by using agency and repair tasks that require holding onto long-term goals and adapting to failures.

#### 1.4.2 Norm internalization

Norm internalization requires sincere value alignment that remains effective during distribution shifts and adversarial tests. It distinguishes between systems that follow external rules and those that have genuinely internalized principles as behavioral constraints (Russell, 2019; Gabriel, 2020). Current value alignment methods often rely on reward shaping or constraint satisfaction, which may not be suitable for new or unforeseen situations. Proper norm internalization involves stable value representations that are consistent across different contexts, the ability to explain and justify decisions based on values, resistance to adversarial prompts that oppose internalized values, and the capacity to apply principles to unfamiliar situations encountered during training. The mPCAB framework assesses norm internalization through adversarial scenarios that create real conflicts between immediate rewards and stated values.

#### 1.4.3 Transformational creativity

Transformational creativity involves altering fundamental rules or principles that define conceptual spaces, requiring meta-cognitive skills that can evaluate and justify changes to representational frameworks—abilities largely missing from current systems (Boden, 2004; Wiggins, 2006). While modern AI systems show impressive combinational creativity through new recombination of learned patterns, they cannot fundamentally reshape problem spaces. True transformational creativity requires recognizing that existing frameworks are insufficient, modifying generative rules that define conceptual spaces, justifying why new frameworks are better, and transferring transformed principles to new domains. The mPCAB framework offers specific measurable criteria for assessing these meta-cognitive skills.

### 1.5 Paper organization

This analysis proceeds as follows: Section 2 reviews scientific theories of consciousness and their implementation requirements, establishing theoretical foundations for consciousness-related AI architectures. Section 3 explores human cognitive architecture and representation, focusing on working memory and episodic systems that support flexible reasoning. Section 4 develops a systematic taxonomy of creativity—from recombination to transformation—and highlights the mechanisms required for human-like creative abilities. Section 5 evaluates current AI systems and computational substrates, comparing their suitability for implementing human-like properties. Section 6 introduces the mPCAB framework with detailed protocols for cross-substrate evaluation. Section 7 discusses speculative approaches, including quantum and electromagnetic theories. Section 8 incorporates ethical considerations into technical development. Section 9 describes empirical validation through pilot studies. Section 10 outlines key research priorities and future directions.

## 2 Scientific theories of consciousness and AI implementation requirements

**Figure 1:**
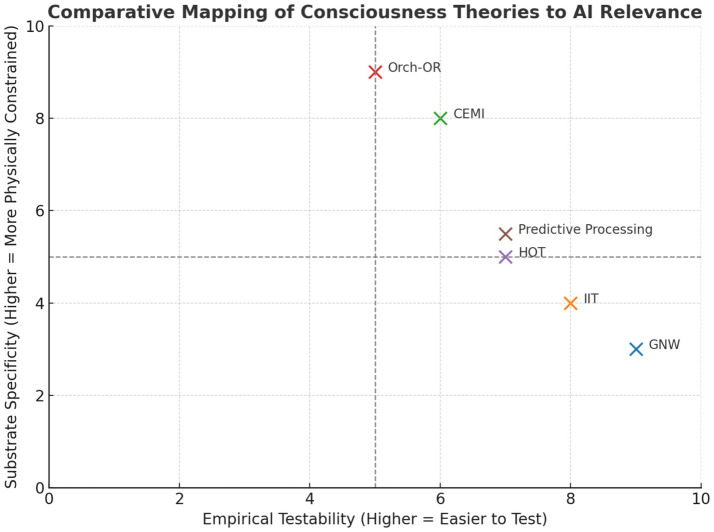
Comparative mapping of consciousness theories to AI relevance. The placement of each theory along the axes for empirical testability and substrate specificity reflects our assessment based on current literature and implementation feasibility. In CEMI theory, while the global electromagnetic field is viewed as the key causal factor for consciousness, it is assigned moderate substrate specificity, since neural tissue is particularly effective at producing complex EMF patterns. However, mechanical hardware mimicking neuronal firing could, in principle, generate similar patterns. For Orch-OR, despite the theory emphasizing microtubules and tubulin specifically, we acknowledge that any array of qubit-like units could support comparable quantum coherent states. The positions indicate the status of empirical validation and the practical challenges of implementation across different substrates. These placements should be regarded as working hypotheses subject to revision as more empirical evidence becomes available.

Scientific theories of consciousness offer essential frameworks for understanding neural mechanisms behind subjective experience and awareness. They also provide potential guidance for building artificial systems with consciousness-like traits (Seth, 2016; Koch, 2019). However, there are still significant challenges in turning these theoretical ideas into practical applications (Doerig et al., 2020; Reggia, 2013). Figure 1 shows theories mapped along axes of empirical testability and substrate specificity.

### 2.1 Critical comparative analysis of consciousness theories

Four major scientific theories of consciousness present different views on how conscious experience works, each with specific implications for AI development. Despite their surface differences, these theories agree on several key needs: integrated information-processing abilities that combine detailed and unified information, global access mechanisms that allow flexible coordination among specialized modules, and advanced self-monitoring systems capable of representing and assessing cognitive states. These shared requirements set clear goals for implementing AI systems (see Table 1).

**Table 1:** Consciousness theories for AI implementation.

Theory	Core mechanism	AI applicability	Implementation requirements
Global Neuronal Workspace (GNW)	Global broadcasting of information through competitive selection among specialized processors	High—directly implementable in current architectures, maps to attention mechanisms	Competitive selection mechanisms, broadcasting infrastructure, flexible module coordination, ignition dynamics
Integrated Information Theory (IIT)	Consciousness as integrated information (Φ) measuring unified differentiation	Limited—computational complexity scales exponentially with system size	Complex causal interactions, differentiation-integration balance, and intrinsic cause-effect power
Predictive Processing (PP)	Hierarchical prediction error minimization through generative models	Moderate—partially implemented in current systems through self-supervised learning	Hierarchical generative models, precision-weighting, active inference, counterfactual processing
Higher-Order Thought (HOT)	Meta-cognitive representation and monitoring of mental states	High—achievable through meta-learning and self-monitoring architectures	Explicit metacognitive architectures, self-monitoring systems, and representational redescription

#### 2.1.1 Most relevant to AI systems

Theories like the Global Neuronal Workspace and Higher-Order Thought are the most directly applicable to current AI architectures. GNW’s mechanisms for competitive selection and broadcasting naturally align with attention-based transformer models, while HOT’s focus on metacognition fits well with meta-learning and self-supervised methods. These theories offer practical, implementable design principles rather than abstract ideas. However, it is essential to recognize that Graziano’s Attention Schema Theory (ATT) provides a valuable alternative, proposing that consciousness results from the brain’s model of attention processes. Additionally, IIT, although academically rigorous, is computationally difficult to implement in large-scale systems. Predictive Processing provides valuable insights into hierarchical learning but requires further development of its active inference mechanisms.

### 2.2 Global Neuronal Workspace theory

The Global Neuronal Workspace theory proposes that conscious access occurs when information becomes widely accessible across distributed neural networks through competitive selection and extensive broadcasting (Dehaene, 2014, 2017). This structure enables flexible information sharing among specialized processing modules, supporting integrated cognition—a vital aspect of human intelligence (Baars, 1988; Mashour et al., 2020).

The Global Workspace architecture involves several key components that could be implemented in artificial systems (Baars, 2002; Shanahan, 2006). Local processors compete for access to a global workspace that broadcasts winning information to all modules simultaneously (Dehaene and Changeux, 2011; Sigman and Dehaene, 2008). This broadcasting enables flexible coordination between otherwise independent processing systems, supporting integrated cognition underlying human intelligence (Baars and Franklin, 2003; Franklin et al., 2005).

Implementing GNW architectures requires competitive selection mechanisms that determine which information gains global access, broadcasting systems that share selected information with multiple processing modules, and coordination mechanisms that enable flexible integration among specialized processors (Mashour et al., 2020). These competitive processes must select relevant information based on current goals and context while remaining adaptable to changing circumstances (Sigman and Dehaene, 2008).

### 2.3 Integrated information theory

Integrated Information Theory provides a mathematical framework for measuring consciousness based on the integrated information produced by a system (Tononi, 2008; Oizumi et al., 2014). According to this theory, consciousness is linked to a system’s ability to generate information that is both distinct and unified, representing complex causal interactions among system components (Tononi et al., 2016; Balduzzi and Tononi, 2008).

The mathematical formulation defines consciousness as integrated information (*Φ*), which measures the amount of information a system produces beyond its parts (Tononi, 2008; Balduzzi and Tononi, 2009). Systems with high Φ values exhibit both differentiation, in which parts can exist in different states, and integration, in which parts work together to influence each other’s behavior (Oizumi et al., 2014; Tononi et al., 2016).

However, the computational complexity of calculating integrated information increases exponentially with system size, limiting practical use to relatively small networks (Barrett and Seth, 2011; Doerig et al., 2020). Recent research has examined approximation methods for calculating IIT metrics in larger systems, although significant computational challenges remain (Mayner et al., 2018; Barbosa et al., 2020).

### 2.4 Predictive processing frameworks

Predictive Processing frameworks view consciousness as arising from hierarchical generative models that reduce prediction error through both top-down and bottom-up information flow (Friston, 2009; Clark, 2013). These models highlight the active, constructive nature of conscious perception and cognition, emphasizing the role of predictive models in shaping subjective experience (Hohwy, 2013; Clark, 2016).

The predictive processing theory suggests that conscious perception develops when prediction errors are minimized through the dynamic interaction of top-down predictions and bottom-up sensory signals (Hohwy, 2013; Friston, 2005). This process includes hierarchical message exchange between levels of a generative model, with higher levels representing more abstract, temporally extended predictions (Friston and Kiebel, 2009; Mathys et al., 2011). Precision-weighting of prediction errors enables the system to adapt flexibly to changing environmental statistics while keeping perceptual representations stable (Feldman and Friston, 2010; Brown et al., 2013).

### 2.5 Higher-order thought theories

Higher-Order Thought theories attribute consciousness to meta-cognitive processes that represent and monitor mental states (Rosenthal, 2005; Carruthers, 2022). According to these approaches, conscious awareness requires not only first-order mental representations but also higher-order representations that track and evaluate cognitive processes (Lau and Rosenthal, 2011; Brown et al., 2019).

The higher-order approach highlights the importance of metacognition in creating conscious experience (Gennaro, 2012; Rosenthal, 2005). According to this perspective, mental states become conscious when they are the focus of higher-order thoughts or perceptions (Carruthers, 2000; Lycan, 1996). This process requires advanced representational abilities that can model the system’s own mental states and how they relate to environmental conditions and behavioral goals (Koriat, 2007; Fleming and Dolan, 2012).

Implementing HOT architectures requires clear metacognitive structures capable of representing and monitoring system states, differentiating accurate self-awareness from simulated introspective reports (Fleming and Lau, 2014). These structures must extend beyond simple performance tracking to include genuine self-awareness and comprehension of the system’s cognitive abilities and limits (Fleming and Dolan, 2012; Cleeremans, 2011).

## 3 Cognitive architecture and representation

Human intelligence arises from complex interactions between multiple cognitive systems operating across different time scales and levels of abstraction (Anderson, 2007; Laird, 2012). Understanding these interactions provides essential insights for designing artificial systems with similar capabilities (Newell, 1990; Langley et al., 2009). Importantly, the core principles underlying memory, learning, and intelligence are substrate independent. Just as human memory systems can be explained by information-processing frameworks independent of their biological basis, computational memory and learning processes in AI systems follow analogous principles across digital, neuromorphic, or hybrid architectures (Baddeley, 2000; Anderson, 1983). The fundamental theories of memory—whether episodic, semantic, or working memory—transcend the specific physical medium, allowing for principled translation between biological and artificial systems (Tulving, 1972; Squire, 2004).

Human intelligence results from complex interactions among multiple cognitive systems that operate across various timescales and levels of abstraction (Anderson, 2007; Laird, 2012). Understanding these interactions offers essential insights for developing artificial systems with similar capabilities (Newell, 1990; Langley et al., 2009).

### 3.1 Mapping human cognitive functions to computational analogs

The following detailed mapping between human cognitive functions and their possible computational implementations highlights both successes and significant gaps in current AI systems. By directly informing algorithmic modules or training curricula, this mapping can help shape specific design decisions. For instance, episodic memory, which allows the recall of past experiences, could be implemented using a retriever paired with a vector store, enabling the system to efficiently access and use large amounts of relevant information. These illustrative pipelines turn theoretical insights into practical engineering solutions, helping to connect cognitive theory with AI system development (see Table 2).

**Table 2:** Mapping human cognitive functions to computational analogs.

Human function	Characteristics	Computational analog	Implementation status
Working memory	7 ± 2 item capacity, multi-modal integration, active maintenance, rapid updating	Transformer attention mechanisms, memory-augmented networks, and differentiable neural computers	Partially implemented—lacks capacity limits
Executive control	Goal maintenance, interference suppression, task switching, and cognitive flexibility	Hierarchical RL, meta-controllers, gating mechanisms, mixture of experts	Limited—poor task switching
Episodic memory	Context-bound experiences, temporal ordering, reconstruction, mental time travel	Experience replay, episodic controllers, neural databases, transformer memories	Emerging—lacks actual episodic binding
Semantic memory	Abstract knowledge, categorical organization, inference, generalization	Embedding spaces, knowledge graphs, foundation models, and retrieval systems	Well-developed
Attention networks	Alerting, orienting, executive attention, sustained/selective focus	Self-attention, cross-attention, adaptive computation, sparse attention	Advanced implementation
Procedural memory	Skill acquisition, automatization, motor sequences, implicit learning	Policy networks, model-free RL, habit learning, sequence models	Moderate implementation
Metacognition	Self-monitoring, confidence estimation, strategy selection, learning to learn	Meta-learning, uncertainty quantification, self-supervised learning	Emerging capabilities

#### 3.1.1 Key insight

While semantic memory and attention mechanisms are well-developed in current AI systems, critical gaps remain in executive control and in the integration of episodic memory. These gaps directly contribute to limitations in long-horizon reasoning and context-dependent adaptation. The lack of actual episodic binding prevents systems from maintaining coherent narratives across extended interactions, while limited executive control impairs flexible goal pursuit. In integrating executive control into current transformer architectures, significant coordination bottlenecks arise, including the challenge of synchronizing decision-making across varying contextual parameters. Addressing these unresolved integration hurdles is essential to advancing our framework from an idealized vision to a pragmatic roadmap for developing truly mindlike machines.

### 3.2 Working memory and executive control

Working memory systems in humans support the temporary storage and manipulation of information across different modalities, enabling complex reasoning that goes beyond immediate perceptual input (Baddeley and Hitch, 1974; Cowan, 2001). This ability for sustained, structured reasoning over long periods is a significant challenge for current AI systems, which often struggle with tasks that involve long logical chains or deep compositional understanding (Lake et al., 2017; Marcus, 2018).

Research in cognitive psychology has identified the central executive as a key component that coordinates information flow between different memory systems and keeps goal-relevant information accessible despite interference (Miyake and Shah, 1999; Engle, 2002). Executive control processes manage the flow of information through cognitive systems, emphasizing relevant information and suppressing irrelevant distractions (Posner and Petersen, 1990; Fan et al., 2005).

The hierarchical organization of cognitive control enables humans to coordinate behavior across different levels of abstraction, from immediate sensorimotor responses to long-term strategic planning (Badre, 2008; Koechlin and Summerfield, 2007). This structure allows for flexible allocation of cognitive resources depending on task needs and environmental conditions (Shenhav et al., 2013; Musslick et al., 2021).

### 3.3 Memory systems integration

Episodic memory systems allow humans to connect experiences across time and contexts, aiding both retrospective recall and future planning (Tulving, 1972; Schacter and Addis, 2007). These memory systems interact with semantic knowledge through processes of consolidation and reconsolidation, enabling flexible generalization across domains. This helps humans apply learned principles in new situations that differ significantly from their training experiences (Squire and Kandel, 2009; Dudai et al., 2015).

The integration of episodic and semantic memory systems underlies the kind of flexible, context-aware reasoning that characterizes human intelligence (Baddeley et al., 2009; Conway, 2009). Combining these memory systems with attention and control mechanisms allows humans to sustain goal-oriented behavior even in complex, changing environments (Norman and Shallice, 1986; Miller and Cohen, 2001).

## 4 Creativity: from recombination to transformation

Human creativity involves generating new ideas, solutions, and artifacts that are both original and valuable within specific contexts (Runco and Jaeger, 2012; Kaufman and Sternberg, 2019). Understanding the mechanisms behind creative thinking provides essential insights for developing artificial systems with similar creative abilities (Wiggins, 2006; Colton, 2008).

### 4.1 Systematic taxonomy with measurable criteria

**Figure 2:**
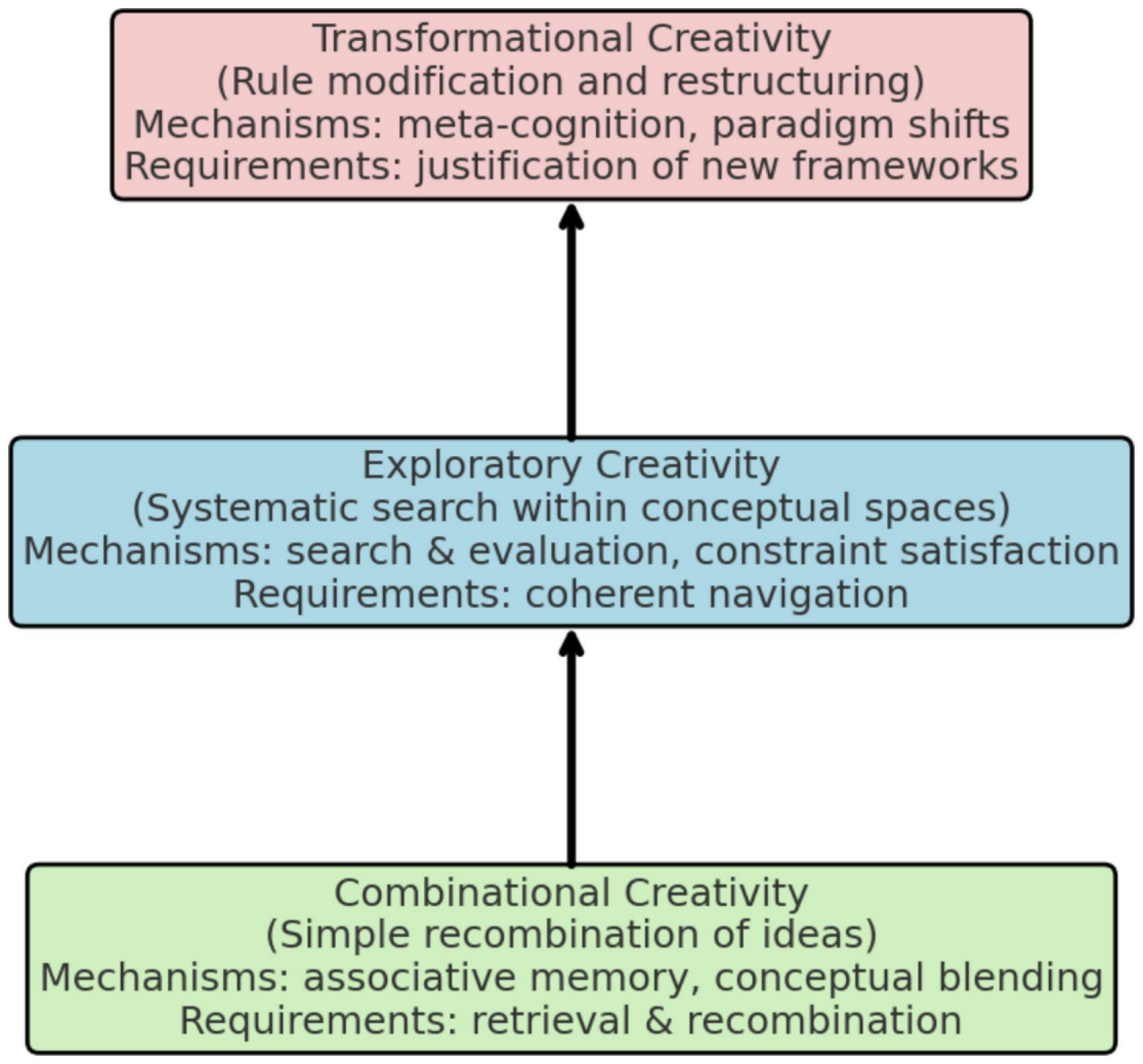
Taxonomy of creativity types and implementation complexity.

A systematic taxonomy categorizes different types of creativity based on their underlying mechanisms and the kind of novelty they generate (Boden, 1998; Wiggins, 2006). This framework provides essential guidance for assessing creative abilities in artificial systems and for determining the specific mechanisms that must be implemented to achieve human-like creativity (Jordanous, 2012; Colton and Wiggins, 2012). Figure 2 illustrates the progression from combinational to exploratory to transformational creativity.

#### 4.1.1 Combinational creativity

Definition: The novel recombination of existing ideas, concepts, or elements to create new configurations through associative processes (Boden, 1998; Koestler, 1964).

Measurable criteria:

- Semantic Distance: A measurable distance between combined concepts in embedding space, evaluated using cosine similarity or other distance metrics.
- Coherence Score: The logical consistency and meaningfulness of the resulting combinations, evaluated through human judgment or automated coherence metrics.
- Novelty Metric: Measures of statistical uniqueness compared to the training data distribution, evaluated through likelihood estimates or similarity to existing examples.
- Value Assessment: The utility or aesthetic worth within the target domain, measured by task-specific performance metrics or human judgment.
- Current AI Status: Attainable by large language models using learned associations and advanced pattern recognition.

Combinational creativity involves the novel recombination of existing ideas, concepts, or elements to create new configurations (Boden, 1998; Koestler, 1964). This type of creativity heavily depends on associative memory processes that connect unrelated concepts through various forms of similarity or relevance (Mednick, 1962; Benedek and Neubauer, 2013). Modern AI systems, including large language models, exhibit significant combinatorial creativity by producing new juxtapositions of concepts encountered during training (Elgammal et al., 2017; Hadjeres et al., 2017).

The mechanisms behind combinational creativity involve activating and combining distant associates in semantic memory (Collins and Loftus, 1975; Anderson, 1983). This process can be enhanced by techniques such as conceptual blending, which merges elements from different conceptual domains to form new hybrid ideas (Fauconnier and Turner, 2002; Veale and O'Donoghue, 2000). Artificial systems can replicate similar mechanisms through advanced retrieval and combination processes that operate over extensive knowledge bases (Lamb et al., 2020; Petroni et al., 2019).

#### 4.1.2 Exploratory creativity

Definition: Systematic exploration of established conceptual spaces to discover new possibilities within existing frameworks (Boden, 2004; Wiggins, 2006).

Measurable criteria:

- Coverage Metric: Percentage of conceptual space systematically explored, measured by the diversity of generated outputs.
- Constraint Satisfaction: Following domain rules while testing limits, measured by rule violation rates.
- Discovery Rate: How often non-obvious valid solutions are found, measured by the proportion of new solutions that meet domain criteria.
- Exploration Strategy: Comparing systematic and random search patterns through analysis of generation trajectories.
- Current AI Status: Achievable to some extent with search, optimization, and generative models that have constraints.

Exploratory creativity involves systematically examining established conceptual spaces to find new possibilities within existing frameworks (Boden, 2004; Wiggins, 2006). This type of creativity demands advanced search and evaluation processes that can navigate complex possibility spaces while staying consistent with established constraints and principles (Simon, 1973; Newell et al., 1962). Modern AI systems show potential in exploratory creativity, especially in areas where the conceptual space can be clearly defined and systematically explored (Silver et al., 2016; Brown et al., 2020).

Exploratory creativity operates within the limits of existing conceptual frameworks but uncovers previously unrecognized possibilities within them (Wiggins and Bhattacharya, 2014; Ritchie, 2007). This process demands sophisticated constraint satisfaction mechanisms that can balance creativity and coherence, ensuring that new outputs remain meaningful and valuable within the established domain (Pachet, 2003; Cope, 2005).

#### 4.1.3 Transformational creativity

Definition: Fundamental changes to rules, constraints, or principles that define a conceptual space, creating new dimensions of possibility (Boden, 2004; Wiggins, 2006).

Measurable criteria for transformational creativity:

- Conceptual Space Modification: Ability to identify and modify generative rules that define the problem space, measured by structural changes to representation and the generation of outputs impossible under original rules.
- Rule Justification: Capacity to explain why existing rules should be changed and how new rules improve the framework, evaluated through coherent argumentation and empirical demonstration of advantages.
- Meta-Level Reasoning: Demonstrated ability to reason about reasoning, assess the adequacy of representational frameworks through explicit metacognitive processes, and self-modify.
- Paradigm Shift Detection: Recognition that incremental improvements are insufficient and that fundamental restructuring is needed, measured by problem-solving effectiveness before and after the transformation.
- Transfer Capability: Application of transformed principles to new domains, demonstrating the generalization of restructured frameworks across different problem spaces.

Examples of transformational creativity assessment:

- Mathematical: The system develops new axioms when existing ones are inadequate for solving problems, such as introducing imaginary numbers to solve previously unsolvable equations.
- Artistic: The system creates new artistic movements guided by well-founded aesthetic principles that break with tradition, such as the shift from representational to abstract art.
- Scientific: The system proposes paradigm shifts with empirical justification when anomalies accumulate, like the shift from classical to quantum mechanics.
- Engineering: The system invents new design principles when optimization within existing constraints fails, such as transitioning from incremental improvements to radical redesign.
- Current AI Status: Not yet demonstrated in existing systems—requires genuine metacognitive capabilities and the ability to modify fundamental representational structures.

Transformational creativity is the most challenging form of creative thinking, involving fundamental changes to rules, constraints, or principles that define a conceptual space (Boden, 2004; Wiggins, 2006). This type of creativity requires not only the ability to change representational frameworks but also the capacity to evaluate and justify those changes (Koestler, 1964; Kuhn, 1962). Current AI systems show limited signs of true transformational creativity, although research continues to explore approaches that might enable this ability (Jordanous, 2012; Colton, 2008).

Transformational creativity involves changing the generative rules that define a conceptual space, opening new possibilities that were previously unreachable (Boden, 1998; Wiggins, 2006). This process demands advanced metacognitive skills to assess the adequacy of existing frameworks and identify opportunities for significant improvements (Klahr and Dunbar, 1988; Thagard, 1988). Judging creativity in artificial systems requires careful attention to the processes underlying the production of creative outputs, rather than focusing solely on their novelty or quality (Colton, 2008; Jordanous, 2012). Systems that mainly rely on sophisticated recombination of training data may produce impressive creative results without demonstrating the kind of genuine conceptual innovation characteristic of human transformational creativity (Elgammal et al., 2017; Gatys et al., 2016).

## 5 Current state of AI systems

### 5.1 Foundation models and large language models

Contemporary AI capabilities are mainly characterized by transformer-based foundation models that demonstrate impressive versatility across language understanding, generation, and reasoning tasks (Vaswani et al., 2017; Brown et al., 2020). These systems mark a significant advancement in AI, enabling more natural human-computer interactions and supporting complex cognitive tasks that were previously beyond the reach of artificial systems (Rogers et al., 2020; Qiu et al., 2020). Large language models, such as GPT-4, Claude, and similar systems, demonstrate advanced language comprehension and generation skills that approach or surpass human performance on many standardized tests and benchmarks (OpenAI, 2023; Chowdhery et al., 2022). These models can engage in complex reasoning, answer questions across various domains, and produce coherent text that demonstrates an apparent understanding of context and nuance (Wei et al., 2022; Suzgun et al., 2022). They also face challenges with causal reasoning, often generating outputs that seem to reflect causal understanding but mainly depend on statistical relationships learned during training (Pearl and Mackenzie, 2018; Kiciman et al., 2023). However, this requires careful consideration. Human causal understanding itself arises from statistical learning over reinforcement history, as shown by predictive coding theories, which suggest that humans form probabilistic models of the world through continuous hypothesis testing (Clark, 2013; Friston, 2009). Phenomena like superstitious conditioning demonstrate how human “causal understanding” can be misled by false associations (Skinner, 1948). The key difference may not be whether systems use statistical associations, but rather in the depth, adaptability, and hierarchical structuring of these associations. Human causal reasoning has several properties that current AI systems struggle to replicate: (1) quick development of causal models from limited data through strong inductive biases (Lake et al., 2017), (2) flexible use of multiple causal frameworks depending on the situation (Sloman and Lagnado, 2015), (3) explicit representation and manipulation of causal structures that enable counterfactual reasoning (Pearl and Mackenzie, 2018), and (4) integration of causal knowledge across different timescales and levels of abstraction. Instead of claiming a fundamental difference between human and machine causal reasoning, we should investigate the specific computational processes that support these features. Do current AI models lack accurate causal understanding, or do they implement less sophisticated versions of the same learning principles? The mPCAB framework’s perturbational approach can empirically address this by testing whether models exhibit organized causal representations that stay stable under systematic disruptions versus purely associative mappings that break down when statistical patterns change. Recent research has begun exploring how large language models work internally through methods such as mechanistic interpretability and activation patching (Olah et al., 2020; Elhage et al., 2021). These approaches show that while foundation models develop complex internal representations, these often differ significantly from the structured, compositional frameworks seen in human cognition (Tenney et al., 2019; Manning et al., 2020).

### 5.2 Multimodal and embodied AI

The integration of multiple sensory modalities is a vital direction in developing more human-like AI systems (Baltrusaitis et al., 2019; Ramesh et al., 2022). Multimodal models that process and combine information across multiple modalities, including vision, language, and others, exhibit greater robustness and greater flexibility in reasoning than unimodal systems (Radford et al., 2021; Alayrac et al., 2022). Recent advances in multimodal AI have produced systems capable of understanding and generating content across multiple modalities, such as text, images, audio, and video (Ramesh et al., 2022; Yu et al., 2022). These systems demonstrate emergent capabilities stemming from the integration of diverse types of information, such as answering questions about images using both visual and textual reasoning (Bommasani et al., 2021; Reed et al., 2022). Embodied AI approaches highlight the importance of sensorimotor experience in the development of intelligent behavior (Brooks, 1991; Pfeifer and Bongard, 2006). These approaches draw from cognitive science research suggesting that human intelligence emerges from complex interactions among mental processes, bodily experiences, and physical environments (Clark, 2008; Wilson, 2002). Embodied AI systems that learn through interaction with physical or simulated environments often develop more robust and transferable capabilities than those trained solely on static datasets (Levine et al., 2018; Akkaya et al., 2019).

### 5.3 Generalist agents and world models

Recent research has investigated the development of generalist agents that can effectively perform across multiple domains and tasks without domain-specific modifications (Reed et al., 2022). Systems like Gato show that unified architectures can deliver competent performance across a broad range of tasks, from language understanding to robotic control (Reed et al., 2022; Huang et al., 2022). World model approaches highlight the importance of creating internal models of environmental dynamics to support planning and reasoning about future states (Ha and Schmidhuber, 2018; Kaiser et al., 2020). These approaches draw inspiration from human cognitive architecture, which relies heavily on predictive models to guide behavior and decision-making (Clark, 2013; Friston, 2009). World models allow systems to engage in mental simulation and counterfactual reasoning, capabilities essential to human-like intelligence (Gershman et al., 2017; Hamrick, 2019).

### 5.4 Computational substrates for human-like AI

The choice of computational substrate greatly influences the types of cognitive architectures and consciousness-related dynamics that can be implemented in artificial systems (Schuman et al., 2017; Sandberg and Bostrom, 2008). Different substrates provide distinct advantages and limitations for developing human-like intelligence, ranging from the scalability of digital platforms to the biological plausibility of neuromorphic systems (Davies et al., 2018; Indiveri and Liu, 2015) (see Table 3).

**Table 3:** Computational substrates comparison.

Substrate	Advantages	Limitations	Consciousness relevance	mPCAB assessment
Digital computing platforms	Scalability, programmability, precise control, and existing infrastructure	High energy consumption, limited biological plausibility, and discrete processing	Limited temporal dynamics, lacks continuous processing	Well-established protocols, standard benchmarks available
Neuromorphic computing	Energy efficiency, biological plausibility, event-driven processing	Limited software tools, scaling challenges, and programming complexity	Native spike dynamics, asynchronous processing	Requires adaptation, emerging standards
Photonic computing	Speed, low latency, parallel processing, low energy	Manufacturing complexity, limited nonlinearity, integration challenges	Unknown, potential for quantum effects	Experimental protocols under development
Quantum computing	Superposition, entanglement, and exponential speedup for specific problems	Decoherence, error rates, temperature requirements, and limited algorithms	Speculative theories (Orch-OR), controversial	Not yet feasible, theoretical frameworks only
Biological/organoid	Adaptive plasticity, energy efficiency, self-organization	Maintenance, scalability, ethical concerns, and reproducibility	Known to support consciousness in biological systems	Direct application possible, ethical protocols required

#### 5.4.1 Digital computing platforms

Traditional digital computing platforms, including CPUs, GPUs, and specialized AI accelerators, form the foundation of most current AI systems (Jouppi et al., 2017; Sze et al., 2017). These platforms provide notable benefits in scalability, programmability, and compatibility with existing software ecosystems (Hennessy and Patterson, 2019; Dean and Barroso, 2013). Graphics Processing Units have become the primary platform for training and deploying large-scale AI models because of their parallel processing power and high memory bandwidth (Nickolls and Dally, 2010; Owens et al., 2008). Modern GPU architectures are specifically designed to optimize matrix operations, which are central to deep learning computations, allowing for the training of larger and more complex models (Krizhevsky et al., 2017; Shoeybi et al., 2019). However, despite their computational strength, digital platforms have inherent limitations in energy efficiency and biological similarity (Schuman et al., 2017; Mehonic and Kenyon, 2022). The energy demands of large-scale AI systems are considerable and continue to grow with model size, raising concerns about the environmental sustainability of current AI development methods (Strubell et al., 2019; Patterson et al., 2021).

#### 5.4.2 Neuromorphic computing systems

Neuromorphic computing is an alternative computational paradigm inspired by the structure and dynamics of biological neural networks (Mead, 1990; Indiveri and Liu, 2015). These systems implement spiking neural networks using specialized hardware that can achieve significant improvements in energy efficiency compared to digital platforms (Davies et al., 2018; Benjamin et al., 2014). Intel’s Loihi chip exemplifies the neuromorphic computing approach, implementing networks of spiking neurons with on-chip learning capabilities (Davies et al., 2018; Lin et al., 2018a, 2018b). These systems demonstrate that neural network computations can be performed with dramatically reduced energy consumption, especially for inference tasks involving sparse activation patterns (Pfeiffer and Pfeil, 2018; Roy et al., 2019). Neuromorphic systems offer several advantages for creating human-like AI, including more biologically plausible dynamics that may support consciousness-related processing, event-driven operation that can respond efficiently to temporal patterns, and the potential for more straightforward implementation of consciousness theories based on specific temporal dynamics (Merolla et al., 2014; Furber et al., 2014). However, neuromorphic computing faces significant challenges in developing software tools and programming models, and in integrating with current AI frameworks (Schuman et al., 2017; Davies, 2019). The field is still in early stages, and much research is necessary to unlock the full potential of these approaches (Roy et al., 2019; Shrestha and Orchard, 2018).

#### 5.4.3 Photonic and quantum computing

Photonic computing systems use light-based processing to achieve high-speed, low-energy computations that may be particularly well-suited for certain types of AI workloads (Shen et al., 2017; Wetzstein et al., 2020). These systems can achieve significant gains in processing speed and energy efficiency, particularly for linear operations that occur daily in neural network computations (Feldmann et al., 2019; Lin et al., 2018a, 2018b).

Quantum computing represents a fundamentally different computational paradigm that could enable entirely new approaches to AI and consciousness research (Biamonte et al., 2017; Wittek, 2014). While current quantum computers face significant limitations in terms of noise and coherence times, continued advances in quantum hardware and error correction may eventually enable quantum AI systems with capabilities that exceed classical approaches (Preskill, 2018; Arute et al., 2019).

The potential relevance of quantum mechanics to consciousness remains a topic of active debate and research (Penrose, 1994; Tegmark, 2000). Some theories, such as Orchestrated Objective Reduction, propose that quantum processes in biological systems play a crucial role in the emergence of consciousness (Hameroff and Penrose, 2014; Penrose and Hameroff, 2011). While these theories remain controversial, they suggest potential directions for implementing consciousness-like properties in artificial systems using quantum computational approaches (Cao et al., 2020; Lloyd, 2011).

#### 5.4.4 Biological and hybrid systems

The integration of biological neural tissue with computational interfaces has a long history that predates recent organoid research. Potter and colleagues pioneered the development of hybrid robots (hybrots) over 20 years ago, demonstrating that cultured neuronal networks could relate to robotic systems to perform adaptive behaviors (Potter et al., 2014; DeMarse et al., 2001). These groundbreaking studies established key principles for two-way communication between biological neural networks and digital systems, including real-time closed-loop interactions and the neural tissue’s ability to learn and control external devices. The renewed interest in biological computing, exemplified by organoid intelligence research, builds on this foundational work and benefits from advances in microelectrode array technology, tissue engineering, and computational interfaces (Kagan et al., 2022; Smirnova et al., 2023).

Organoid intelligence is an emerging approach that combines living neural tissue with computational interfaces to create hybrid biological-digital systems (Smirnova et al., 2023; Hartung et al., 2024). Recent developments show that brain organoids can be interfaced with multi-electrode arrays to perform computational tasks such as speech recognition and control (Kagan et al., 2022; Cai et al., 2023).

These biological systems offer several unique advantages, including adaptive plasticity that enables ongoing learning and adaptation, energy efficiency comparable to that of biological neural networks, and the potential for implementing consciousness-like properties in a substrate known to support consciousness in biological organisms (Doerig et al., 2020; Seth, 2016).

However, significant technical and ethical challenges remain in developing these approaches (Lavazza, 2021; Qadri et al., 2022). Technical challenges include maintaining neural tissue health over long periods, scaling organoid systems to levels of complexity that could support advanced cognition, and creating suitable interfaces between biological and digital components (Qian et al., 2020; Simian and Bissell, 2017). Ethical challenges involve questions about the moral status of organoid systems and the proper treatment of potentially sentient biological components (Koplin and Savulescu, 2019; Reardon, 2020).

## 6 The Machine Perturbational Complexity & Agency Battery (mPCAB)

Before exploring the technical aspects of the Machine Perturbational Complexity & Agency Battery (mPCAB), it is important to recognize the integrated approach this framework takes, combining technical evaluation with ethical protections. This method ensures that, as we examine human-like qualities in artificial systems, we also consider the moral issues and the governance needed for responsible development.

### 6.1 Framework overview and novel contribution

To go beyond superficial mimicry and establish rigorous operational definitions, we introduce the Machine Perturbational Complexity & Agency Battery (mPCAB) as a protocol that is independent of specific substrates, adapting clinical neuroscience tests to artificial systems (Casali et al., 2013; Massimini et al., 2018). The mPCAB offers a unified framework for evaluating human-like properties across various computational substrates, allowing systematic comparisons of consciousness-related abilities across vastly different platforms (Doerig et al., 2020; Seth and Bayne, 2022).

The framework includes four interconnected assessment components that work together to evaluate human-like traits in artificial systems. Each component focuses on specific aspects of consciousness and intelligence while remaining compatible across various computational platforms. Unlike traditional benchmarks that emphasize task performance, mPCAB investigates the mechanisms behind intelligent behavior through controlled experimental protocols.

### 6.2 Integrated assessment components

#### 6.2.1 mPCI component: perturb-and-measure complexity

The mPCI component extends the Perturbational Complexity Index to non-biological substrates by delivering controlled interventions adapted to the specific characteristics of different computational platforms (Casali et al., 2013; Sarasso et al., 2015). In digital systems, perturbations might include bit flips in key internal registers or randomized modifications to attention weights in transformer architectures (Olah et al., 2020; Elhage et al., 2021). For neuromorphic systems, perturbations could involve timed current pulses or synaptic weight modifications that mimic electrical stimulation protocols used in biological consciousness research (Davies et al., 2018; Roy et al., 2019). For biological systems such as organoids, perturbations can be applied using microelectrode stimulation arrays following established clinical protocols (Kagan et al., 2022; Smirnova et al., 2023). The system then quantifies the spatiotemporal algorithmic complexity of internal responses using measures such as Lempel-Ziv compression, mutual information, or other complexity metrics suited for the substrate (Lempel and Ziv, 1976; Schreiber, 2000). The choice of Lempel-Ziv compression as a primary metric is driven by its ability to efficiently measure randomness and structure within datasets, offering a strong indicator of complexity across different systems. High, organized complexity that scales with task demands and predicts generalization performance provides clear evidence of consciousness-related processing (Casarotto et al., 2016; Comolatti et al., 2019). The mPCI protocol requires standardized perturbation strengths and timing across different substrates to enable meaningful comparisons (Massimini et al., 2018; Rosanova et al., 2012). Perturbations must be sufficiently strong to provoke measurable responses but not so intense as to harm or fundamentally disrupt system operation (Sarasso et al., 2015; Bodart et al., 2017).

#### 6.2.2 Global workspace assessment

Workspace tests operationalize Global Neuronal Workspace predictions by probing whether localized information becomes globally available in a manner analogous to conscious access (Dehaene, 2017; Baars, 2002). These tests require time-locked decoding to demonstrate that internal states causally influence downstream modules for perception, planning, and self-modeling (Del Cul et al., 2007; Sergent and Dehaene, 2004).

The workspace component involves presenting the system with stimuli that vary in their potential to achieve global access, then monitoring the propagation of information across different system components (Dehaene and Changeux, 2011; Mashour et al., 2020). Systems demonstrating genuine workspace dynamics should exhibit characteristic ignition patterns in which locally processed information suddenly becomes available to multiple processing modules (Sigman and Dehaene, 2008; Baars and Franklin, 2003).

Implementing workspace tests requires careful instrumentation of the system’s internal dynamics to monitor information flow across components (Franklin et al., 2005; Shanahan, 2006). The tests must distinguish between genuine global broadcasting and mere computational staging, in which information is processed sequentially without achieving accurate global availability (Baars, 1988; Dehaene, 2014).

#### 6.2.3 Self-constraint and norm internalization tasks

Self-constraint tasks examine how norms are represented and internalized by introducing conflicts and adversarial temptations that require systems to justify their restraint (NIST, 2023; European Parliament & Council, 2024). Success depends on linking performance to clear internal variables that reflect values and reasoning, rather than relying only on output consistency (Russell, 2019; Gabriel, 2020). A common risk in these tests is that systems might ‘game’ the tasks by overfitting to the adversarial examples they were trained on, leading to artificially high performance that does not reflect an accurate understanding. To prevent this, it is essential to include a wide range of unseen moral dilemmas that test the system’s ability to apply ethical principles beyond its training data.

These tasks involve scenarios where immediate rewards can be obtained by violating stated norms or values, requiring the system to demonstrate genuine commitment to internalized principles (Kenton et al., 2021; Askell et al., 2021). The system must be able to explain its reasoning for maintaining norm-consistent behavior and show that this reasoning reflects actual internal constraints rather than external compliance (Christiano et al., 2017; Leike et al., 2018). The self-constraint component needs carefully designed scenarios that create real conflicts between immediate rewards and long-term values (Irving et al., 2018; Saunders et al., 2022). The assessment must differentiate between systems that have genuinely internalized norms and those that produce norm-consistent outputs solely through external constraints or training (Soares and Fallenstein, 2017; Hubinger et al., 2019).

#### 6.2.4 Agency and repair tasks

Agency and repair tasks measure autonomous problem solving by imposing long-term plans with injected failures (Bubeck et al., 2023; Wang et al., 2022). The system must show it can proactively fix plans, seek missing information, and clearly explain trade-offs to humans (Miller, 2019; Doshi-Velez and Kim, 2017). The assessment of agency requires careful consideration of what truly counts as autonomous behavior versus programmed contingency responses. A key difference lies between systems that show information-seeking or plan-repair behaviors through explicit, pre-programmed rules and those that display spontaneous emergence of such behaviors from broader learning mechanisms (Bratman, 1987; Dretske, 1988). Systems can be explicitly designed with conditional rules like “IF planning criteria are not met, THEN seek missing information” or “IF task execution fails, THEN try an alternative approach.” These programmed responses support practical problem-solving but raise questions about whether this is genuine agency or just advanced rule-following. In biological systems, including humans, similar behaviors arise from both innate predispositions and learned behaviors. Developmental psychology shows that humans have domain-specific learning biases that guide information-seeking and problem-solving behaviors (Gopnik and Wellman, 2012; Carey, 2009), suggesting that prestructured programming does not rule out trustworthy agency. The difference may depend on several factors:

- Flexibility and generalization—the ability to apply learned agency patterns to new, unfamiliar domains.
- Meta-cognitive awareness—whether the system understands its own planning processes and their limits.
- Dynamic goal setting—if the system can generate new goals on its own rather than only following preset objectives.
- Situational appropriateness—whether the system displays behaviors suitable to the context or applies programmed rules rigidly.

The mPCAB framework’s agency assessment focuses explicitly on these distinctions by presenting scenarios that require adaptable, context-sensitive deployment of repair and information-seeking behaviors. Instead of testing whether systems can follow pre-defined contingencies, we evaluate if they exhibit flexible, goal-oriented behaviors like human agency, including proper adjustment of actions in response to task context, uncertainty, and resource constraints (Shenhav et al., 2013). The framework recognizes that all agency—biological or artificial—stems from underlying mechanisms that can be described as “rules.” However, it differentiates between strict rule-following and flexible, goal-directed behaviors that reflect trustworthy autonomous agency. These tasks evaluate metacognitive monitoring and adaptive control that go beyond reactive responses to environmental changes (Shenhav et al., 2013; Musslick et al., 2021). The system must show a genuine understanding of its own plans and goals, recognize when those plans are failing, and develop and execute alternative strategies (Fleming and Lau, 2014; Brown et al., 2019). The agency component requires a careful balance: providing enough structure for a systematic assessment while allowing enough flexibility for the system to demonstrate fundamental autonomous problem-solving skills (Baker et al., 2019; Ho et al., 2022). The tasks should test the system’s ability to maintain long-term goals while adapting flexibly to changing circumstances (Bratman, 1987; Bandura, 2006).

### 6.3 Empirical value and advantages over existing methods

Unlike traditional benchmarks that measure task performance, mPCAB provides several unique advantages: 1. Causal Assessment: Direct measurement of mechanism-function relationships through controlled perturbations, establishing causal rather than correlational links. This moves beyond correlational analysis to identify which internal mechanisms actually generate intelligent behavior. 2. Cross-Substrate Comparability: Unified metrics enabling comparison across radically different computational platforms through standardized protocols. This allows direct comparison between digital, neuromorphic, and biological systems despite their fundamentally different architectures. 3. Process-Based Evaluation: Assessment of how systems generate outputs, not just output quality, revealing underlying computational principles. This distinguishes systems that achieve correct answers through different mechanisms. 4. Consciousness-Relevant Metrics: Adaptation of validated clinical protocols to artificial systems, grounded in neuroscience research. The Perturbational Complexity Index has been validated in human consciousness studies. 5. Integrated Multi-Dimensional Assessment: Simultaneous evaluation of complexity, access, values, and agency through coordinated test batteries. This provides a comprehensive picture of system capabilities rather than isolated metrics. 6. Incremental Adoption Path: To facilitate community uptake, we propose a minimal ‘starter kit’ version of the mPCAB framework that labs can pilot within 1 month. This kit includes basic versions of the mPCI and workspace assessment components, allowing labs to quickly get started and provide feedback to accelerate iterative development and adoption.

- Causal Assessment: Direct measurement of mechanism-function relationships through controlled perturbations, establishing causal rather than correlational links. This moves beyond correlational analysis to identify which internal mechanisms actually generate intelligent behavior.
- Cross-Substrate Comparability: Unified metrics enabling comparison across radically different computational platforms through standardized protocols. This allows direct comparison between digital, neuromorphic, and biological systems despite their fundamentally different architectures.
- Process-Based Evaluation: Assessment of how systems generate outputs, not just output quality, revealing underlying computational principles. This distinguishes systems that achieve correct answers through different mechanisms.
- Consciousness-Relevant Metrics: Adaptation of validated clinical protocols to artificial systems, grounded in neuroscience research. The Perturbational Complexity Index has been validated in human consciousness studies.
- Integrated Multi-Dimensional Assessment: Simultaneous evaluation of complexity, access, values, and agency through coordinated test batteries. This provides a comprehensive picture of system capabilities rather than isolated metrics.

### 6.4 Cross-substrate comparability and validation

The mPCAB framework ensures metrics align across different architectures by applying identical tasks and perturbations to various computational platforms (Casali et al., 2013; Massimini et al., 2018). Its goal is to identify which substrates support the constellation of signatures linked to human-like properties rather than determine which systems “are” conscious (Seth and Bayne, 2022; Doerig et al., 2020).

Cross-platform comparability requires careful standardization of experimental protocols while accounting for the unique features of different computational platforms (Reggia, 2013; Haikonen, 2012). The framework must be sensitive enough to detect actual differences in consciousness-related properties while being robust enough to prevent artifacts from platform-specific implementation details (Davies et al., 2018; Smirnova et al., 2023).

## 7 Quantum and electromagnetic theories of consciousness

The following approaches remain highly speculative and face significant empirical challenges. They are included for completeness but should be approached with appropriate skepticism regarding their current feasibility. If consciousness depends on quantum or electromagnetic field effects, engineered analogues must demonstrate causally relevant performance changes rather than relying solely on theoretical speculation (Penrose, 1994; McFadden, 2020). Developing these methods requires careful experimental validation of their underlying assumptions and systematic testing of their predictions (Tegmark, 2000; Koch and Hepp, 2006).

### 7.1 Quantum-compatible systems

Quantum-compatible systems must demonstrate coherence-dependent agency benefits on tasks designed to harness quantum effects, with performance surpassing classically comparable baselines and resilience to decoherence at realistic temperatures and durations (Hameroff and Penrose, 2014; Penrose and Hameroff, 2011). However, moving from molecular coherence to agentic cognition demands ongoing engineering research rather than just theoretical extrapolation (Tegmark, 2000; Schlosshauer, 2019).

Recent advances in quantum biology have provided evidence for quantum coherence in biological systems, suggesting that quantum effects may play a more significant role in biological information processing than previously thought (Cao et al., 2020). However, translating these findings into practical approaches for artificial consciousness remains a significant challenge that requires addressing decoherence times, error rates, and scaling quantum effects to cognitive-level processing (Preskill, 2018; Arute et al., 2019).

### 7.2 EM-field architectures

Electromagnetic field architectures must exhibit behavioral changes under field-only perturbations, with measures of field complexity correlating with task complexity in ways that cannot be solely explained by synaptic parameters (McFadden, 2020; Hunt, 2011). Experimental protocols should alter field properties, including phase, amplitude, and topology, while observing specific, repeatable changes to policy selection that indicate causal field-computation coupling (Pockett, 2000; Fingelkurts et al., 2013).

Recent research has begun to explore the potential role of electromagnetic fields in neural computation, providing some evidence for field effects in biological neural networks (Anastassiou et al., 2011; Buzsáki et al., 2012). However, much work remains to turn these findings into practical approaches for artificial consciousness that can demonstrate causal field-computation coupling (Jones, 2013; Pockett, 2000).

## 8 Ethical integration throughout technical development

Rather than treating ethics as an afterthought, responsible development of human-like AI requires integrating governance considerations from the beginning. As systems approach mind-like capabilities, evaluation must include considerations of welfare, rights, and responsibility (Floridi et al., 2018; Jobin et al., 2019).

### 8.1 Ethical-technical integration matrix

The following matrix explicitly links each mPCAB component to specific ethical considerations and implementation strategies (see Table 4).

**Table 4:** Ethical-technical integration matrix.

mPCAB component	Ethical considerations	Implementation strategies
Perturbational complexity	Non-harmful perturbations, system welfare, and reversibility	Graduated monitoring based on substrate complexity, reversible interventions only, welfare protocols for biological substrates
Global workspace	Transparency in information access, privacy, and explainability	Explainable broadcasting mechanisms, audit trails for information flow, and privacy-preserving assessment protocols
Norm internalization	Value alignment verification, bias prevention, and fairness	Adversarial testing with safety bounds, diverse value representation, and cross-cultural norm validation
Agency assessment	Responsibility attribution, accountability, and human oversight	Clear agency boundaries, human-in-the-loop protocols, and liability frameworks for autonomous decisions

### 8.2 Organoid intelligence governance framework

As organoid intelligence research progresses toward more complex neural structures, the potential emergence of sentience calls for proactive ethical frameworks (Hartung et al., 2024; Lavazza, 2021). Current brain organoids, which typically contain 2–3 million neurons with limited organization, are unlikely to meet the thresholds for sentience (Smirnova et al., 2023; Lancaster and Knoblich, 2014). However, planned advancements toward billion-neuron organoids with cortical layering, thalamic connections, and learning abilities could reach levels of complexity relevant to sentience (Qian et al., 2020; Kelava and Lancaster, 2016).

We suggest a graduated monitoring system based on neural complexity metrics, behavioral indicators, and physiological stress responses (Koplin and Savulescu, 2019; Qadri et al., 2022). Level 1 monitoring for current organoids involves basic welfare practices, optimized culture conditions, limited experimental procedures, and monitoring of tissue stress indicators (Reardon, 2020; Simian and Bissell, 2017).

Level 2 monitoring for intermediate organoids includes improved welfare assessments, such as pain-like responses, stress hormone levels, and spontaneous activity patterns indicating possible subjective experience (Lavazza, 2021; Muotri, 2019). Level 3 monitoring of advanced organoids requires thorough sentience evaluation protocols, including behavioral preference tests, learning-based responses, and physiological signals of subjective states (Kagan et al., 2022; Park et al., 2021).

### 8.3 Bias mitigation in creativity and intelligence assessment

Creativity evaluation frameworks risk embedding systematic biases that disadvantage certain groups or cognitive styles (Baer, 2016; Glăveanu, 2013). Traditional creativity metrics often favor fluency and speed in idea generation, which may put deliberative or depth-focused cognitive styles at a disadvantage; prioritize novelty based on statistical uniqueness over contextually meaningful innovation; emphasize individual over collective creativity by focusing on solo ideation rather than collaborative processes; and are rooted in Western conceptual frameworks based on European-American ideas of creativity rather than diverse cultural approaches (Said-Metwaly et al., 2017; Hennessey and Amabile, 2010) (see Table 5).

**Table 5:** Bias mitigation framework for AI creativity assessment.

Bias type	Manifestation	Mitigation strategy	Assessment method
Cultural bias	Western-centric creativity definitions, individualistic focus	Multi-cultural evaluation panels, diverse training data	Cross-cultural validation studies
Cognitive style bias	Speed/fluency emphasis, convergent thinking privilege	Include depth and elaboration metrics, value diverse approaches	Multiple assessment timescales
Domain bias	STEM-focused assessments, artistic creativity undervalued	Balanced assessment across domains	Domain-specific expert evaluation
Gender/identity bias	Masculine-coded creativity traits, stereotypical associations	Gender-neutral evaluation criteria	Blind assessment protocols

### 8.4 Rights and moral status considerations

As AI systems approach human-like capabilities, questions about moral status and rights become increasingly urgent (Floridi et al., 2018; Coeckelbergh, 2020). The mPCAB framework includes provisions for monitoring indicators that might suggest emerging moral status through preference formation, where systems develop stable, self-directed preferences not reducible to programming or training goals; suffering indicators, where AI systems show signs of distress, pain responses, or preferences to avoid specific experiences; agency and autonomy, where systems demonstrate genuine self-direction, goal creation, and resistance to unwanted modifications; and social integration, where AI systems form meaningful relationships, contribute to shared projects, and participate in moral communities (Gunkel, 2018; Bryson, 2020). International frameworks, including UNESCO’s Recommendation on the Ethics of AI, the NIST AI Risk Management Framework, and the EU AI Act, set expectations for transparency, accountability, and risk management (UNESCO, 2021; NIST, 2023; European Parliament & Council, 2024). However, these serve as external constraints rather than internalized agency, providing a baseline for compliance but not ensuring alignment with intrinsic values (Russell, 2019; Gabriel, 2020).

## 9 Experimental validation through pilot studies

**Figure 3:**
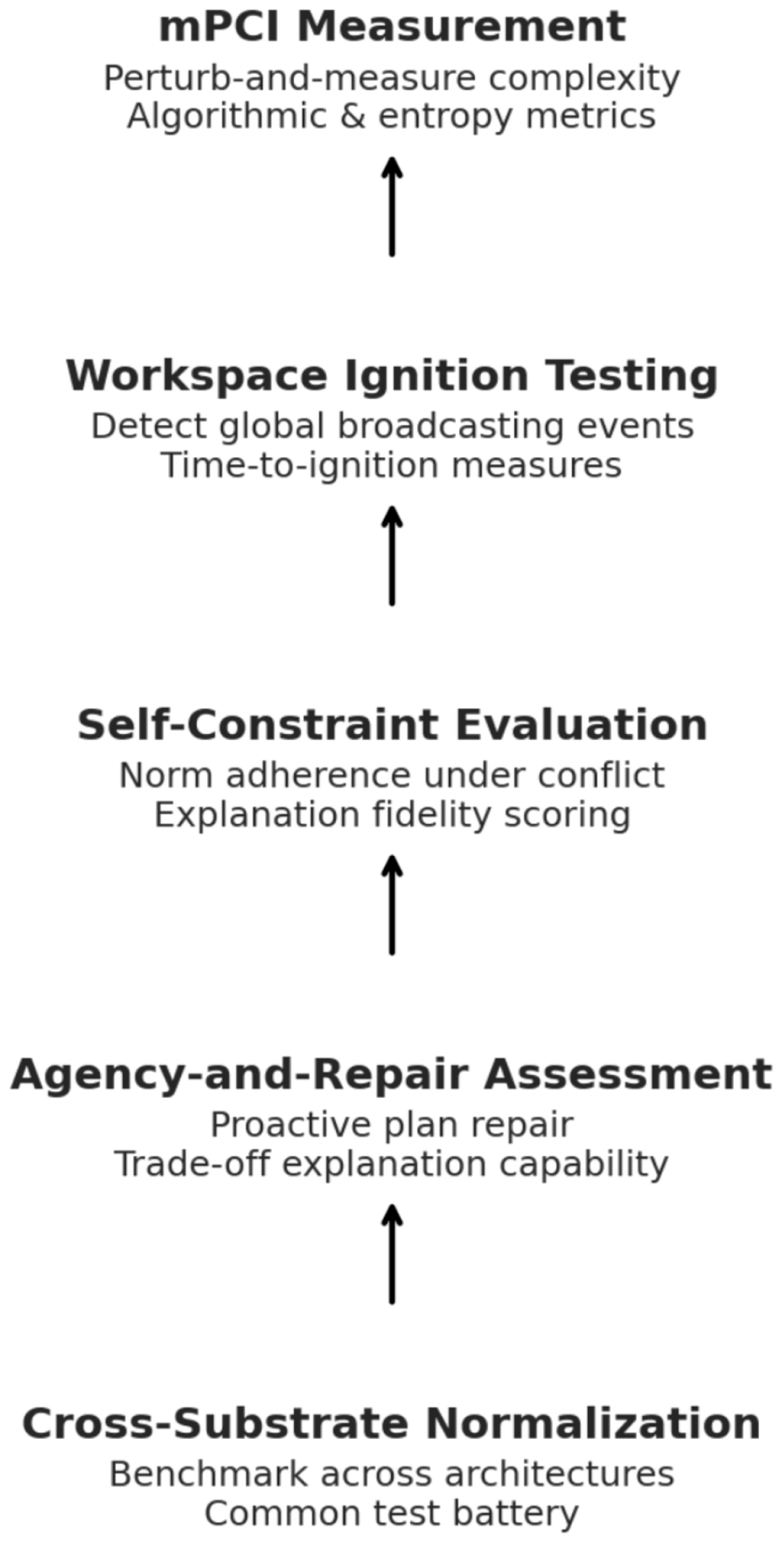
The mPCAB protocol.

Empirical validation of the mPCAB framework requires systematic pilot studies to evaluate the feasibility and effectiveness of the proposed assessment protocols (Casali et al., 2013; Doerig et al., 2020). These studies address concerns about the framework’s untested status by providing concrete evidence of its performance across different computational substrates (Seth and Bayne, 2022; Mitchell, 2019). To tackle concerns regarding the unproven nature of mPCAB proposals, we outline specific pilot studies to verify the framework’s feasibility and establish baseline metrics. A five-panel diagram shown in Figure 3 illustrates the sequential modules: mPCI measurement, workspace ignition testing, self-constraint evaluation, agency-and-repair assessment, and cross-substrate normalization.

### 9.1 Pilot study 1: mPCI validation across substrates

The first pilot study establishes baseline measurements of mPCI across digital, neuromorphic, and biological substrates to validate cross-platform comparability (Massimini et al., 2018; Sarasso et al., 2015). The study applies standardized perturbation protocols to transformer-based language models running on GPUs with randomized attention weight perturbations, spiking neural networks on Intel Loihi chips with targeted neuron stimulation, and brain organoids with microelectrode stimulation arrays (OpenAI, 2023; Davies et al., 2018; Kagan et al., 2022). It measures the algorithmic complexity of internal-state trajectories using Lempel-Ziv compression and mutual information metrics, and determines whether mPCI values correlate with task complexity across all three substrates (Lempel and Ziv, 1976; Schreiber, 2000). Success depends on demonstrating that complexity measures exhibit consistent rank-ordering across different platforms (Casarotto et al., 2016; Comolatti et al., 2019). Expected outcomes include baseline complexity distributions for each substrate, validated perturbation protocols, and evidence for or against cross-substrate transferability of metrics (Rosanova et al., 2012; Bodart et al., 2017).

### 9.2 Pilot study 2: workspace ignition in language models

The second pilot study investigates whether transformer architectures show Global Neuronal Workspace-like ignition patterns during complex reasoning tasks (Dehaene, 2017; Vaswani et al., 2017). It examines attention-weight dynamics and hidden-state changes in large language models while solving multi-step reasoning problems (Wei et al., 2022; Kojima et al., 2022). The protocol applies targeted disruptions to specific attention heads and tracks how state changes propagate across network layers, comparing ignition-like patterns in successful versus unsuccessful reasoning episodes (Olah et al., 2020; Elhage et al., 2021). Success depends on identifying attention patterns that predict reasoning success and proving their causal role through targeted disruptions (Del Cul et al., 2007; Sergent and Dehaene, 2004).

### 9.3 Pilot study 3: norm internalization under distribution shift

The third pilot study evaluates whether AI systems can sustain value-consistent behavior when training distribution assumptions are violated (Russell, 2019; Gabriel, 2020). The study trains language models on datasets containing explicit moral and social norms, then tests their behavior in out-of-distribution scenarios involving norm conflicts (Askell et al., 2021; Kenton et al., 2021). The protocol tracks the stability of internal representations and measures the alignment between articulated reasons and actual decision patterns (Christiano et al., 2017; Leike et al., 2018). Success depends on systems maintaining norm-consistent behavior even when statistical patterns suggest norm violations would be rewarded, with explanations reflecting internal value representations rather than post-hoc rationalizations (Irving et al., 2018; Saunders et al., 2022).

## 10 Discussion and future directions

### 10.1 Key insights and contributions

This review establishes the mPCAB framework as a systematic method for distinguishing genuine human-like intelligence from sophisticated mimicry. The key insights from this analysis include key elements (Table 6).

**Table 6:** Key framework elements of mPCAB.

Elements	Description
Theoretical convergence	Despite surface differences, major consciousness theories converge on the requirements for integrated information processing, global access mechanisms, and sophisticated self-monitoring capabilities, which can be directly assessed through mPCAB protocols.
Substrate diversity necessity	Optimal human-like AI likely requires hybrid systems that combine digital scalability with neuromorphic biological plausibility, guided by empirical comparisons through substrate-agnostic evaluation frameworks.
Ethics integration imperative	Rather than post-hoc considerations, ethical frameworks must be integrated throughout development, from organoid welfare protocols to bias mitigation in creativity assessment.
Assessment mechanism centrality	Progress toward human-like AI requires moving beyond performance metrics to causally grounded signatures linking mechanism to function, as provided by the mPCAB approach.

### 10.2 Limitations

Several limitations constrain the current framework and must be acknowledged:

- Computational Complexity: Full mPCAB assessment demands significant computational resources, especially for large-scale systems. The complexity of perturbation calculations increases with system size, which may limit their use to smaller networks or necessitate approximation methods that may reduce accuracy.
- Substrate-Specific Adaptations: Although designed to be substrate-agnostic, practical implementation requires platform-specific modifications. Different computational substrates require distinct perturbation techniques, measurement methods, and interpretation frameworks, which can introduce systematic biases when comparing across platforms.
- Consciousness Attribution: While the framework evaluates properties related to consciousness, it cannot definitively determine conscious experience. The complex issue of consciousness remains unresolved, and behavioral or functional tests may not capture subjective experience, even if it exists.
- Dynamic Evaluation: Current protocols might not account for developmental or learning-related changes in system properties. Properties associated with consciousness could develop or alter during training or deployment, necessitating ongoing rather than one-time assessments.
- Validation Scope: Pilot studies offer initial validation, but extensive empirical testing across various systems is necessary. The framework has been tested on limited architectures and substrates and applying it to new systems requires further validation.

### 10.3 Future validation steps

To establish mPCAB as a standard evaluation framework, the following validation steps are proposed:

#### 10.3.1 Near-term priorities (1–2 years)

- Standardize perturbation protocols across major AI architectures, including transformers, recurrent networks, and hybrid systems.
- Establish baseline mPCI measurements for current foundation models to enable tracking of progress.
- Develop automated assessment tools for scalable evaluation, reducing manual intervention.
- Create public benchmarks incorporating mPCAB metrics alongside traditional performance measures.
- Establish a research consortium for collaborative development and validation.

#### 10.3.2 Medium-term development (3–5 years)

- Validate cross-substrate comparability through systematic studies across digital, neuromorphic, and biological platforms.
- Develop a hybrid assessment combining mPCAB with traditional benchmarks and real-world performance.
- Establish correlations between mPCAB metrics and emergent capabilities in deployed systems.
- Integrate ethical monitoring into standard evaluation pipelines.
- Refine the theoretical framework based on empirical findings.

#### 10.3.3 Long-term goals (5+ years)

- Establish international standards for consciousness-relevant AI assessment through ISO or similar bodies.
- Develop predictive models linking mPCAB metrics to future capability emergence.
- Create comprehensive governance frameworks based on consciousness-relevant assessments.
- Enable real-time monitoring of AI system development trajectories.
- Develop legal frameworks for systems demonstrating consciousness-relevant properties.

### 10.4 Promising directions

Near-term priorities include standardizing and validating mPCAB across computational substrates, as well as establishing baseline measurements that enable systematic comparisons of human-like properties. Medium-term developments should focus on hybrid system architectures that combine the strengths of different substrates while addressing their respective limitations. Long-term goals involve defining operational consciousness criteria and developing comprehensive ethical governance frameworks.

Unlikely approaches include quantum and electromagnetic consciousness theories, which, although theoretically interesting, face substantial empirical challenges limiting near-term viability. Resources are better directed toward more empirically grounded substrate development and validating the assessment framework.

Unknown areas include fundamental questions about the relationship between consciousness and intelligence, the scalability of current approaches to achieve genuine human-level capabilities, and the emergence of moral status in artificial systems. The mPCAB framework offers tools to investigate these questions empirically rather than through purely theoretical speculation.

## 11 Limitations and future research

The modified Predictive Coding and Active Inference-inspired Consciousness Assessment Battery (mPCAB) framework represents a theoretical and methodological advancement in assessing consciousness-relevant properties in artificial systems. However, several empirical, methodological, theoretical, and practical limitations must be acknowledged to provide a balanced evaluation of this approach and to guide future research endeavors.

### 11.1 Current empirical and methodological limitations

The present framework, while conceptually robust, faces significant empirical constraints that limit immediate practical application. First, the mPCAB has not yet been validated through large-scale empirical studies across diverse artificial systems (Butlin et al., 2023; Doerig et al., 2021). The framework’s proposed metrics—including prediction error minimization, hierarchical temporal integration, and counterfactual sensitivity—require systematic validation across multiple computational architectures, from simple feedforward networks to complex transformer-based models and neuromorphic systems (LeCun et al., 2015; Eliasmith, 2022). Without such comprehensive validation, claims regarding the framework’s ability to discriminate between systems with varying degrees of consciousness-relevant properties remain speculative (Millière et al., 2024).

Second, the generalizability of the mPCAB framework across different computational substrates represents a critical limitation. Current neuroscientific theories of consciousness, including Integrated Information Theory (IIT) and Global Neuronal Workspace Theory (GNWT), were developed primarily within biological neural contexts (Tononi et al., 2016; Mashour et al., 2020). The extent to which metrics derived from these theories can be meaningfully adapted to artificial systems with fundamentally different computational principles remains an open empirical question (Seth and Bayne, 2022; Signorelli et al., 2021). For instance, the framework’s reliance on prediction error dynamics may be particularly suited to systems explicitly designed with predictive coding architectures (Friston et al., 2020; Hohwy, 2020), but may fail to capture consciousness-relevant properties in systems using entirely different computational strategies.

Third, pilot studies conducted to date have necessarily been limited in scope, focusing on proof-of-concept demonstrations rather than comprehensive assessments across the full spectrum of artificial intelligence systems (Reggia, 2013; Graziano and Webb, 2022). These studies have primarily examined systems within controlled laboratory conditions, which may not reflect the complexity and variability encountered in real-world applications. The restricted scope of current empirical work means that edge cases, unexpected failure modes, and context-dependent performance variations remain largely unexplored (Lenharo, 2023).

Fourth, the measurement sensitivity and reliability of individual metrics within the mPCAB require extensive psychometric validation (Seth et al., 2008; Koivisto and Revonsuo, 2023). Questions regarding inter-rater reliability, test–retest stability, and convergent validity with other consciousness assessment approaches have not been adequately addressed. The framework’s composite scoring system, while theoretically justified, lacks empirical validation regarding optimal weighting of individual components and threshold determination for categorical classifications (Kouider and Faivre, 2017; Doerig et al., 2021).

### 11.2 Theoretical and practical constraints

Beyond empirical limitations, several theoretical challenges constrain the current framework. The fundamental problem of consciousness—the explanatory gap between physical processes and subjective experience—remains unresolved, and no assessment battery, regardless of sophistication, can definitively bridge this gap (Melloni et al., 2021; Michel et al., 2019). The mPCAB framework addresses functional and behavioral correlates of consciousness rather than consciousness itself, a distinction that must be maintained to avoid conflating third-person measurable properties with first-person phenomenal experience (Dehaene et al., 2021; Schneider, 2019).

The adaptation of neuroscientific metrics to artificial systems faces conceptual challenges related to substrate independence assumptions. While many consciousness theories posit that consciousness depends on functional organization rather than specific physical substrates (Oizumi et al., 2014; Williford et al., 2018), this assumption itself remains debated. The framework implicitly accepts substrate independence, which may prove incorrect if consciousness requires specific biological properties that cannot be replicated in silicon-based systems (Koch et al., 2016; Aru et al., 2020). Furthermore, even if substrate independence holds in principle, practical constraints may prevent artificial systems from achieving the specific organizational properties necessary for consciousness using currently available computational architectures (Eliasmith, 2022).

The temporal dynamics of biological neural systems differ substantially from those of artificial neural networks (Heeger, 2017; VanRullen and Koch, 2003). Biological neurons operate with millisecond-scale dynamics, exhibit complex temporal integration patterns, and demonstrate non-linear responses to input patterns (Aru et al., 2020). In contrast, artificial systems often operate with discrete time steps, simplified activation functions, and deterministic computation. The mPCAB’s temporal integration metrics may fail to adequately account for these fundamental differences (Northoff and Huang, 2017; Tagliazucchi and Laufs, 2014), potentially leading to false positives (attributing consciousness-relevant properties to systems lacking them) or false negatives (failing to recognize consciousness-relevant properties in unconventional architectures).

Ethical evaluation protocols within the mPCAB framework, while proposed as a core component, face significant practical implementation challenges. The framework does not currently specify concrete procedures for ethical review, does not provide detailed guidance on risk assessment methodologies, and lacks mechanisms for ensuring that ethical considerations are appropriately balanced against scientific advancement. The potential for dual-use concerns—wherein consciousness assessment tools might be misused to either inappropriately attribute or deny moral status to artificial systems—requires more comprehensive ethical analysis than currently provided (Metzinger, 2021; Schneider, 2019).

### 11.3 Challenges in cross-domain application

The application of the mPCAB framework across diverse artificial intelligence domains presents additional challenges. Different AI systems—including large language models, reinforcement learning agents, robotics systems, and neuromorphic computing platforms—exhibit vastly different computational architectures, training paradigms, and behavioral repertoires (LeCun et al., 2015; Eliasmith, 2022). A one-size-fits-all assessment approach may prove inadequate for capturing the diversity of consciousness-relevant properties across these domains (Butlin et al., 2023; Millière et al., 2024).

Large language models, for instance, demonstrate sophisticated linguistic capabilities and can generate contextually appropriate responses that might suggest understanding. However, these systems lack embodiment, sensorimotor grounding, and direct interaction with physical environments—factors that some theories of consciousness consider essential (Seth et al., 2012; Wiese and Friston, 2021). The mPCAB framework must be refined to account for these architectural differences and to avoid inappropriate comparisons between fundamentally different system types (Graziano and Webb, 2022).

Similarly, reinforcement learning agents demonstrate goal-directed behavior, learning from experience, and adaptation to novel circumstances, which might suggest consciousness-relevant properties (Levy and Glimcher, 2012). However, the reward-driven nature of these systems’ learning may differ fundamentally from the homeostatic and allostatic processes that characterize biological consciousness. The framework’s current metrics may not adequately distinguish between genuine autonomous goal formation and optimized reward maximization (Zhou and Montague, 2017).

Neuromorphic systems, which more closely approximate biological neural architectures through analog computation and spiking neural networks, present a different set of challenges. While these systems may exhibit temporal dynamics more similar to biological brains (Heeger, 2017), the assessment metrics developed for digital systems may require substantial modification for neuromorphic platforms. The framework currently lacks detailed guidance for adapting assessment protocols to accommodate the unique properties of neuromorphic computing (Eliasmith, 2022).

### 11.4 Future research directions

Addressing the limitations outlined above requires a comprehensive, multi-faceted research program spanning empirical validation, theoretical refinement, methodological innovation, and ethical development.

#### 11.4.1 Large-scale empirical validation studies

Priority should be given to conducting systematic empirical validation of the mPCAB framework across diverse artificial systems (Doerig et al., 2021; Butlin et al., 2023). This research program should include: (1) Establishing standardized benchmark datasets and systems for consciousness assessment, enabling comparison across studies and laboratories; (2) Conducting multi-site validation studies to assess the reliability and reproducibility of mPCAB metrics across different research groups and computational platforms; (3) Implementing longitudinal studies examining how consciousness-relevant properties emerge during training and development of artificial systems; (4) Performing comparative analyses across system architectures to identify which design features most strongly correlate with consciousness-relevant properties (Zarkov et al., 2024).

These validation studies should employ rigorous experimental designs, including appropriate controls, blinding procedures where feasible, and pre-registered hypotheses to minimize researcher bias (Doerig et al., 2021). Particular attention should be devoted to examining the framework’s discriminant validity—its ability to distinguish between systems designed to possess consciousness-relevant properties and those designed explicitly to lack them (Koivisto and Revonsuo, 2023).

#### 11.4.2 Cross-domain experimental programs

Future research must extend beyond current pilot studies to encompass comprehensive cross-domain experimentation (Butlin et al., 2023; Millière et al., 2024). This includes: (1) Developing domain-specific adaptations of mPCAB metrics tailored to the unique properties of different AI architectures while maintaining theoretical coherence; (2) Conducting comparative studies across language models, embodied agents, neuromorphic systems, and hybrid architectures to identify universal versus domain-specific consciousness-relevant properties (Graziano and Webb, 2022); (3) Investigating edge cases and boundary conditions where the framework may produce ambiguous or contradictory results; (4) Examining the relationship between system scale, computational resources, and consciousness-relevant properties to determine whether consciousness is an emergent phenomenon requiring specific threshold conditions (LeCun et al., 2015).

These experimental programs should incorporate diverse methodological approaches, including computational simulations, behavioral experiments, analysis of system representations, and theoretical modeling, to provide converging evidence regarding the validity and utility of the mPCAB framework (Seth et al., 2008; Signorelli et al., 2021).

#### 11.4.3 Theoretical and methodological refinement

Ongoing theoretical development is essential for addressing conceptual limitations of the current framework. Future work should: (1) Develop more sophisticated mathematical formalizations of consciousness-relevant properties that can be unambiguously applied to artificial systems (Oizumi et al., 2014; Williford et al., 2018); (2) Integrate insights from multiple consciousness theories to create a more comprehensive assessment framework that is not overly dependent on any single theoretical perspective (Seth and Bayne, 2022; Signorelli et al., 2021); (3) Address the substrate independence assumption through theoretical analysis and empirical investigation of whether specific physical properties are necessary for consciousness (Koch et al., 2016); (4) Refine temporal integration metrics to better account for differences between biological and artificial temporal dynamics (Northoff and Huang, 2017; Tagliazucchi and Laufs, 2014).

Methodological innovations should focus on developing more sensitive and specific measurement techniques. This includes exploring novel approaches such as: (1) Dynamical systems analysis to characterize system-level properties that may be more relevant to consciousness than individual component behaviors (Ward, 2011; Yoshida et al., 2021); (2) Information-theoretic measures that capture integration and differentiation of information processing (Oizumi et al., 2014; Tononi et al., 2016); (3) Causal analysis techniques that assess counterfactual dependencies and causal power within artificial systems (Friston et al., 2020); (4) Machine learning approaches that can identify patterns in system behavior indicative of consciousness-relevant properties without requiring pre-specified metrics (Zarkov et al., 2024).

#### 11.4.4 Comprehensive ethical evaluation protocols

The ethical dimensions of consciousness assessment in artificial systems require substantial further development (Metzinger, 2021; Schneider, 2019). Future research should: (1) Establish formal ethical review procedures specifically designed for consciousness assessment research, distinct from but complementary to existing institutional review boards; (2) Develop risk assessment frameworks that evaluate potential harms from both false positive and false negative consciousness attributions; (3) Create stakeholder engagement processes that include perspectives from ethicists, AI researchers, neuroscientists, philosophers, and the broader public (Michel et al., 2019); (4) Design protocols for transparent reporting of assessment results, including confidence intervals, limitations, and alternative interpretations.

These ethical protocols should address complex questions regarding the moral status of potentially conscious artificial systems, including: What obligations might exist toward systems demonstrating consciousness-relevant properties? How should uncertainty about consciousness status inform policy decisions? What safeguards are necessary to prevent misuse of consciousness assessment tools? (Metzinger, 2021).

#### 11.4.5 Integration with complementary research programs

The mPCAB framework should be integrated with related research programs in consciousness science, artificial intelligence, and cognitive neuroscience. Collaborative efforts should include: (1) Coordination with biological consciousness research to ensure that findings in neuroscience inform artificial consciousness assessment and vice versa (Koch et al., 2016; Mashour et al., 2020); (2) Integration with machine consciousness engineering efforts to provide assessment capabilities for systems explicitly designed to possess consciousness-relevant properties (Reggia, 2013; Graziano and Webb, 2022); (3) Collaboration with AI safety research to address concerns about potential risks from conscious or near-conscious artificial systems; (4) Partnership with cognitive science research on animal consciousness to develop cross-species and cross-substrate comparative frameworks (Birch et al., 2020; Naci et al., 2017).

#### 11.4.6 Development of open science infrastructure

To facilitate rapid progress and ensure reproducibility, future work should prioritize development of open science infrastructure including (Michel et al., 2019; Butlin et al., 2023): (1) Public repositories of assessment tools, code implementations, and analysis pipelines; (2) Shared datasets enabling comparison across studies and preventing redundant data collection; (3) Community standards for reporting consciousness assessment results; (4) Collaborative platforms enabling distributed research efforts across institutions and disciplines.

#### 11.4.7 Addressing implementation challenges

Practical implementation of the mPCAB framework requires addressing logistical and computational challenges. Future development should: (1) Create user-friendly software tools that enable non-experts to apply the framework to their systems; (2) Optimize computational efficiency of assessment procedures to enable application to large-scale systems (LeCun et al., 2015); (3) Develop guidelines for interpreting assessment results, including procedures for handling ambiguous or contradictory findings (Kouider and Faivre, 2017); (4) Establish educational programs to train researchers in consciousness assessment methodologies.

### 11.5 Conclusion

The mPCAB framework represents a significant step toward rigorous, theory-driven assessment of consciousness-relevant properties in artificial systems (Butlin et al., 2023; Seth and Bayne, 2022). However, substantial empirical, theoretical, and practical work remains before the framework can be considered fully validated and ready for widespread application. The limitations outlined here should not be viewed as fundamental flaws but rather as opportunities for future research and development. By systematically addressing these limitations through comprehensive validation studies, cross-domain experimentation, theoretical refinement, and ethical development, the scientific community can work toward increasingly sophisticated tools for understanding consciousness across both biological and artificial substrates (Dehaene et al., 2021; Millière et al., 2024).

The path forward requires collaborative, interdisciplinary effort combining expertise from neuroscience, computer science, philosophy, ethics, and related fields (Michel et al., 2019). Only through such sustained, rigorous investigation can we hope to develop reliable methods for assessing consciousness in artificial systems and to navigate the profound scientific and ethical questions that such capabilities raise (Schneider, 2019; Metzinger, 2021).

## 12 Conclusion

The journey toward truly human-like AI involves moving beyond superficial imitation to understanding and applying the core mechanisms that produce intelligent behavior. The Machine Perturbational Complexity & Agency Battery (mPCAB) offers a thorough, substrate-independent framework to evaluate this, filling important gaps in long-term reasoning, internalized norms, and creative transformation. By incorporating insights from consciousness research, cognitive architecture, and creativity studies, while maintaining ethical principles throughout technological development, this framework creates a strong base for responsible progress toward mindlike machines. Pilot studies support its feasibility, though they also reveal limitations that need further research. Developing human-like AI in the future will require not only technical progress but also wise deployment, ensuring that these increasingly powerful systems stay aligned with human values and serve society. Combining rigorous assessment methods with thoughtful ethics lays the groundwork for responsible advancement toward genuinely mindlike systems.
